# The Effectiveness of Acupuncture in the Treatment of Frozen Shoulder: A Systematic Review and Meta-Analysis

**DOI:** 10.1155/2020/9790470

**Published:** 2020-09-25

**Authors:** Eyal Ben-Arie, Pei-Yu Kao, Yu-Chen Lee, Wen-Chao Ho, Li-Wei Chou, Hsin-Ping Liu

**Affiliations:** ^1^Graduate Institute of Acupuncture Science, China Medical University, Taichung 40402, Taiwan; ^2^Division of Thoracic Surgery, Department of Surgery, China Medical University Hospital, Taichung 40402, Taiwan; ^3^Department of Acupuncture, China Medical University Hospital, Taichung 40402, Taiwan; ^4^Department of Public Health, China Medical University, Taichung 40402, Taiwan; ^5^Department of Physical Medicine and Rehabilitation, China Medical University Hospital, Taichung 40402, Taiwan; ^6^Department of Physical Therapy and Graduate Institute of Rehabilitation Science, China Medical University, Taichung 40402, Taiwan; ^7^Department of Rehabilitation, Asia University Hospital, Taichung 40402, Taiwan

## Abstract

**Background:**

Frozen shoulder (FS) is associated with pain, reduced range of motion (ROM), and shoulder function. The condition occurs in 2–5% of the population, and it is especially common around the age of 50 years. FS symptoms will recover after 1–4 years. Many patients turn to acupuncture in order to alleviate the FS symptoms.

**Objective:**

In this review, we will investigate the efficiency of acupuncture as a FS treatment.

**Methods:**

A literature search of acupuncture and FS-related keywords was performed in the following databases: PubMed, Cochrane Library, Embase, and Web of Science. Thirteen publications were included for a systematic review, and a meta-analysis was done using the following measurements: visual analogue scale (VAS) for pain, Constant-Murley Shoulder Outcome Score (CMS) for shoulder function, and active shoulder ROM including flexion, abduction, and external rotation. The Cochrane Collaboration's risk of bias tool and quality of evidence GRADE recommendations and STRICTA 2010 were used to grade the included publications.

**Results:**

A meta-analysis on VAS pain score showed significant pain reduction, restoring CMS shoulder function, and flexion ROM in favor of acupuncture versus the control. In external rotation and abduction ROM, a meta-analysis was not significant. The most used acupoints are Jian Yu (LI15) and Jian Liao (TB14).

**Conclusions:**

The results indicate that acupuncture could be safe and effective for pain reduction, restoring shoulder function, and restoring flexion ROM for FS patients in the short term and midterm. However, the level of evidence was very low. More high-quality and longer studies are needed in order to robust the evidence.

## 1. Introduction

Frozen shoulder (FS), also called “adhesive capsulitis” or “periarthritis,” is a condition that occurs in 2–5% of the population, and it is especially common around the age of fifty [1]. The condition affects the shoulder by causing an unexplained pain, stiffness, and limitation in the active and passive range of motion (ROM) in two or more planes and can lead to a progressive loss of shoulder function. The pain will usually increase at night and can affect sleep quality [1, 2]. Females will have a 58% greater risk to suffer from this condition [2]. FS is characterized by three phases: freezing phase, frozen phase, and the thawing phase. The time duration for FS to completely heal varied from 1 to 4 years [3, 4]. Up to 15% of FS patients will suffer from a long-term disability [1, 5]. The highest risk factor for FS is diabetes mellitus; other risk factors are related to any kind of local trauma (proximal humeral fractures or rotator cuff tears), invasive procedure, thyroid disease, dyslipidemia, prolonged immobilization, and stroke [1]. The causes of FS are largely unknown, limited evidence suggests a genetic connection of human leukocyte antigen (HLA) B27 as a risk factor for FS [6]. Arthroscopic observations suggest that inflammation often appears in the anterosuperior joint capsule, axillary fold of the joint capsule, coracohumeral ligament, and in the rotator cuff interval. The inflammation then leads to adhesions and fibrosis [1]. Tissue sample finding indicates an elevation of inflammatory cytokine levels in the subacromial bursa and the anterior capsule area [7]. Patient's history and physical examination usually are sufficient enough in the diagnosis of FS [2]. An important distinction is that the ROM limitation in FS is a pure mechanical limitation that does not occur due to the patient's pain sensation [1].

Until today, there is still disagreement inside the medical community upon which treatment is the most effective in terms of reducing pain and restoring ROM for FS. In a systematic review that was published in 2011, the researchers found strong evidence in favor of laser therapy and steroid injections for pain treatment in the short term [5]. Another review investigated arthroscopic capsular release and showed a significant rapid improvement in shoulder function in the short and long term [8]. None of the studies in the review involved a control group. Patients are usually referred to arthroscopic capsular release after one year of conservative therapy without improvement [8].

Acupuncture has been treating patients for thousands of years. In Jain et al.'s review, the authors moderately recommended the combiunation of acupuncture with physiotherapy (PT) for treating FS, and they reported that acupuncture can reduce pain and improve ROM and shoulder function for patients with FS. Additionally, the review of Jain and Sharma found that electroacupuncture (EA) has a beneficial effect on short-term pain relief [2]. A Cochrane review on “acupuncture for shoulder pain” from 2005 included 9 studies and did not find significant results in favor of acupuncture due to the small sample size and the low studies quality [9]. The last systematic review and meta-analysis concentrated on acupuncture as the treatment method for frozen shoulder were published in 2007 [10]. The review only included six clinical trials and could not recommend acupuncture as a treatment for frozen shoulder due to a small number of studies. Therefore, a more up-to-date review with a larger number of studies is needed. In this review and meta-analysis, we will further investigate the use of acupuncture for FS patients in terms of pain, ROM, and shoulder function.

## 2. Methods

### 2.1. Search Strategy

This review follows the Preferred Reporting Items for Systematic Reviews and Meta-Analyses (PRISMA) statement (see Supplementary Table 1) [11]. PubMed, Embase, Cochrane Library, and Web of Science databases were searched in August 2020. The “keywords” were a combination of “frozen shoulder,” “adhesive capsulitis,” “periarthritis,” “acupuncture,” and “electroacupuncture.” Original papers were searched and collected from January 1999 until August 2020. For PubMed, search strategy limitations were as follows: clinical trial, randomized control trial, and English language, and for Embase, Cochrane Library, and Web of Science (years 2020-1999), no other search limitations were set (for search record, see Supplementary Table 2). This review protocol was registered in “PROSPERO” registration no. CRD42019130352.

### 2.2. Inclusion Criteria

#### 2.2.1. Type of Studies

We included controlled retrospective studies and clinical control trials either randomized or nonrandomized.

#### 2.2.2. Participants

The patients included in the review are primary adhesive capsulitis patients in all three stages of the condition without any limitations of age or demographics. Any other type of adhesive capsulitis patients (such as traumatic, secondary, stroke, and diabetic patients) along with any other shoulder conditions are excluded (in cases when the majority of the study's patients are suffering from secondary adhesive capsulitis).

#### 2.2.3. Interventions and Comparison

Interventions may include common acupuncture interventions including manual acupuncture (MA), EA, and press tack acupuncture. Control interventions included are Western medication, PT intervention (such as interferential electrotherapy (IFE), transcutaneous electrical nerve stimulation (TENS), extracorporeal shockwave therapy (ESWT), shoulder exercises), and sham acupuncture (sham needles or sham acupoints). We also include in the systematic review studies comparing different types of acupuncture treatments.

#### 2.2.4. Outcomes

The primary outcome measurement is pain reduction (critical): through visual analogue scale (VAS) score. Secondary outcome measurements are ROM changes (important) in degrees and shoulder function (important) with ability in daily life (ADL), Constant-Murley Score (CMS), Oxford Shoulder Score (OSS), and Shoulder Pain and Disability Index (SPADI).

### 2.3. Exclusion Criteria

In this review, we excluded studies for duplication, cases in which the abstract or full text is not available, studies that include other shoulder conditions (such as rotator cuff tendinopathy, impingement syndrome, and subacromial bursitis), and also studies that are not in English.

#### 2.3.1. Study Selection

Studies were selected under our study inclusion criteria. A researcher screened the title and abstract of studies. Potential full texts were reviewed by two independent researchers, and any decision on studies inclusion was done after discussion. In case of a decision to exclude a study, the reason for exclusion will be noted (not being a control study, nonrelevance, or not meeting the inclusion criteria).

#### 2.3.2. Data Extraction

Included studies data were tabulated into the following categories: author name, year, number of patients in total, number of patients in each group, interventions information, randomization information, FS duration in each group, number of intervention sessions, study outcome measurements, study results, and study adverse events. In cases of missing data, the reviewers made two attempts to contact the study's authors via email. All quality assessments were reviewed by two independent researchers.

#### 2.3.3. Quality Assessment

For quality assessment, we used the Cochrane Collaboration's risk of bias tool [12]. The tool includes eight questions to assess each study's risk of bias: random sequence generation, allocation concealment, blinding of participants, blinding of study personnel, blinding outcome assessor, incomplete outcome data, selective reporting, or other sources of bias. Other sources of bias were assessed by insufficient reporting, unclear measurement assessment tools, or carryover effects in crossover trials. The Cochrane Collaboration's risk of bias tool has three possible outcomes: high risk of bias, unknown risk of bias, or low risk of bias. For risk of bias graphic table, we used the Review Manager version 5.3 (Copenhagen: The Nordic Cochrane Centre, The Cochrane Collaboration, 2014). Risk of bias was done by three reviewers independently, with Chinese medicine background and academic experience, and any case of disagreement was resolved by discussion.

After the Cochrane Collaboration's risk of bias tool was complete, we graded each study for quality of evidence, using the GRADE recommendations (Grading of Recommendations, Assessment, Development, and Evaluation) for four possible grades: very low, low, moderate, and high [13]. Each clinical trial initial score was high, and one point was reduced in a case when a study fails to complete each of the following segments: lack of allocation concealment, lack of blinding incomplete accounting of patients and outcome events, selective outcome reporting, and other limitations.

Finally, we used an acupuncture reporting checklist, based on “STRICTA 2010 checklist” (Standards for Reporting Interventions in Clinical Trials of Acupuncture) [14]. The “STRICTA 2010 checklist” is part of “CONSORT 2010” (Consolidated Standards of Reporting Trials) quality assessment [15]. The “STRICTA 2010 checklist” is used in order to measure the quality of reporting acupuncture inventions. The checklist contains 17 questions about the manner in which acupuncture was performed. Questions were scored yes = 1 and no = 0, the maximum score is 17, and the lowest score is 0 [16].

#### 2.3.4. Statistical Analysis

A meta-analysis was done using the following measurements: VAS score for pain, CMS shoulder function score, and active shoulder ROM: flexion, abduction, and external rotation. Only studies comparing acupuncture to other interventions were included in the meta-analysis. For statistical analysis, we used the Review Manager version 5.3 (Copenhagen: The Nordic Cochrane Centre, The Cochrane Collaboration, 2014). Continuous data were shown as mean difference (MD) ± standard deviation (SD) with 95% confidence interval (CI). *P* value <0.05 was considered as significant. Heterogeneity tests were done using the chi-squared test and *I*^2^ test. An *I*^2^ score of >50% indicates an existing heterogeneity, and in those cases, we applied a random effect. We applied a fixed effect in cases in which *I*^2^ was lower than 50% (<50%). In case of high heterogeneity, a subgroup analysis was considered (a meta-analysis needs to contain at least two studies). In addition, a subgroup analysis of acupuncture treatment types (such as MA and EA) was done when possible. Also, for each meta-analysis, we rated the strength of evidence (GRADE) in the results with consideration of the included studies design, risk of bias, inconsistency, indirectness, imprecision, and other considerations. Evidence was scored as either high, moderate, low, or very low. For this, we used the GRADEpro online system [17].

## 3. Results

**Figure 1:**
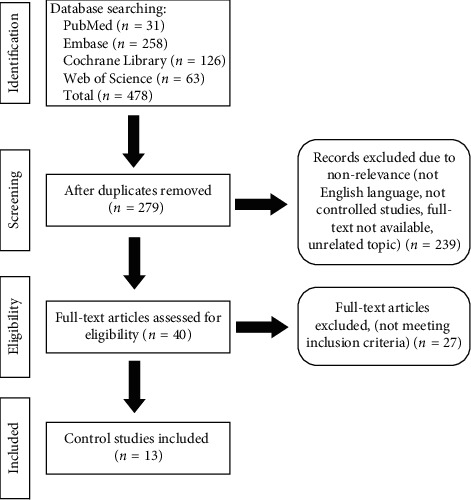
Flowchart of study selection. The flowchart describes the study selection process for included studies in this systematic review and meta-analysis. The flowchart follows the Preferred Reporting Items for Systematic Reviews and Meta-Analyses (PRISMA) statement [11].

A total of 478 studies were found initially in the databases. After removing duplicates (199 duplicates), reviewing abstracts, and removing nonrelevant studies (438 were removed), forty English full-text articles were assessed for eligibility. Out of the 40 full-text articles, 27 articles were excluded due to not being controlled studies, nonrelevance, or not meeting the inclusion criteria. 13 publications were eventually included from January 1999 to August 2020 (Figure 1). A total of 966 FS patients from 13 publications were included in this review. The mean number of patients in each study is 74. All of the 13 studies are clinical controlled trials. Three studies were double-blind clinical trials.

Table 1 represents the main characteristics of the 13 included clinical trials. One study compared MA and scalp acupuncture with ESWT compared to PT exercise and ESWT [19]. One study compared two different MA methods [24]. Seven studies compared acupuncture to PT [18, 19, 22, 23, 26, 27, 30]. Five studies examined the effectiveness of EA [18, 23, 25, 28, 29]. Lo et al. conducted a double-blind study comparing EA with PT to sham EA (sham points) with PT [18]. Shi et al. also examined the effectiveness of moxibustion as a treatment for FS [25]. Lin et al. used a video-based stereophotogrammetry system to compare the ROM of the shoulder after placebo and verum acupuncture [28]. Schroder et al. conducted a double-blind randomized control trial (RCT), using press tack acupuncture needles versus press tack placebo (no needle) to investigate an immediate pain relief [21]. At the completion of the immediate study, they also asked the participants to take part in a year-long single-blind study comparing acupuncture in addition to conservative therapy versus conservative therapy only (mainly oral steroids) [21]. Zhang et al. compared between Ashi points alone to Ashi points and distal MA [20].

### 3.1. Quality Assessment

**Figure 2:**
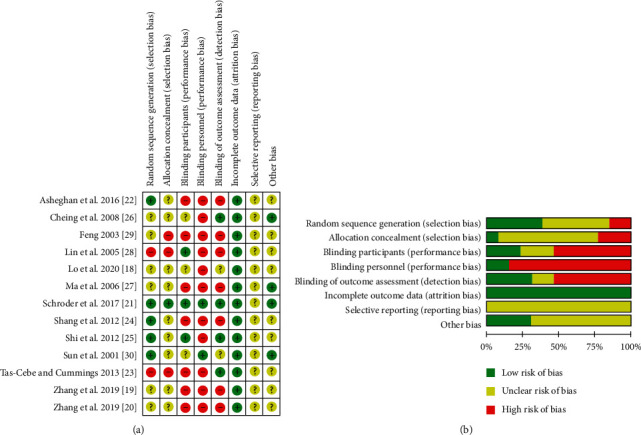
Risk of bias from included studies. (a) Risk of bias summary. Review authors' judgments about each risk of bias item for each included study. (b) Risk of bias graph. Review authors' judgments about each risk of bias item presented as percentages across all included studies.

For quality assessment, the Cochrane Collaboration's risk of bias tool and the quality of evidence grading can be found in Figures 2(a) and 2(b). In the segment of random sequence generation, two studies were rated high risk of bias due to no randomization [23, 28] and six studies did not report the randomization clearly [18–20, 26, 27, 29]. In terms of allocation concealment, three studies did not conceal patients' allocation [23, 27, 28], and most of the studies did not describe clearly their concealment methods [18–20, 22–30]. For participants blinding, only three studies properly blind participants [21, 28, 30]. Most studies did not blind study personnel, due to the difficulty in blinding acupuncturist [18–20, 22–29]. Seven studies failed to blind the outcome assessor [19, 20, 22, 24, 27–29]. All studies completely reported outcome data. For selective reporting, all studies are rated as unclear risk of bias because no study protocol was published prior to the final publication, and in addition, ten studies did not report any adverse events segment [19, 20, 22, 24–30]. In regard to other sources of bias, five studies were rated unclear due to unclear measurement tools and potential biased tools like phone interviews as a follow-up or a carryover effect in crossover trial [19, 20, 24, 25, 28, 29]. It is important to notice that in most acupuncture interventions, it is extremely difficult to blind the acupuncturist, a fact that puts acupuncture studies in an inferior initial position in terms of risk of bias and quality of evidence.

For studies quality of evidence (GRADE), the initial score of most studies was high (*N* = 12) except one study with a very low initial score [29], the final score was very low in one study [29], low in seven studies [18, 23, 24, 26–28, 30], and moderate in four studies [19, 20, 22, 25], and only one study grade remained high [21] (Supplementary Table 3). Finally, in the “STRICTA 2010 checklist” assessment for quality of acupuncture reporting, the maximum score is seventeen and the minimum score is zero (high score indicates proper acupuncture reporting). We have found that nine of the included studies record twelve or more points which are considered as a mediocre score [18, 20, 21, 23–26, 28, 30], and three studies record 10 or less than 10 points, which are considered as a low score [22, 27, 29] (Supplementary Table 4).

### 3.2. Pain

**Figure 3:**
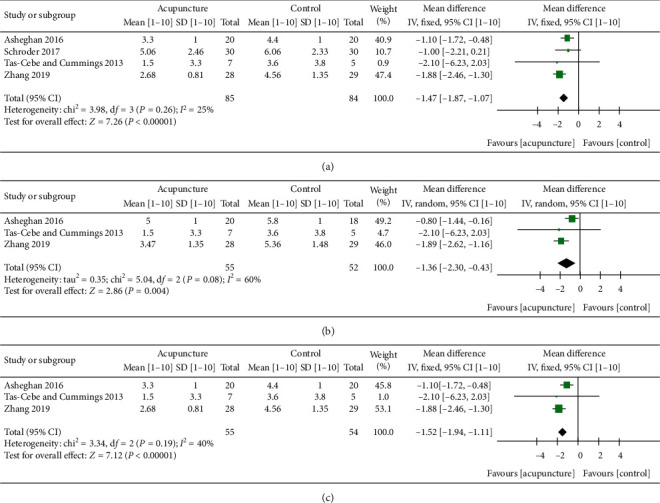
Forest plot of VAS score of MA at different follow-up time course. (a) 0- to 12-week follow-up. Forest plot of comparison: acupuncture (MA + PT, MA + ESWT, press tack needles) versus control (PT + ESWT, PT exercise, press tack placebo). Follow-up time: [21] immediate effect, [23] 6 weeks, [19] 8 weeks, and [22] 12 weeks. (b) 4- to 6-week follow-up. Forest plot of comparison: acupuncture (MA + PT, MA + ESWT) versus control (PT + ESWT, PT exercise). Follow-up time: [19] 4 weeks, [23] 6 weeks, and [22] 6 weeks. (c) 1.5- to 3-month follow-up. Forest plot of comparison: acupuncture (MA + PT, MA + ESWT) versus control (PT + ESWT, PT exercise). Follow-up time: same as in (b). Outcome: VAS score [1–10]. ^*∗*^Schroder et al. 2017 CMS pain subscore 0–15 descending was converted to VAS 1–10 ascending by the following method: 1O − (*M*/1.5)=*M*(VAS), (SD/1.5)=SD(VAS).^*∗*^Sensitivity analysis of Figure 3(b) found that when excluding [19] from the analysis, heterogeneity: tau^2^ = 0.00; chi^2^ = 0.37, d*f* = 1 (*P*=0.54); *I*^2^ = 0%, test for overall effect: *Z* = 2.59 (*P*=0.010).

**Figure 4:**
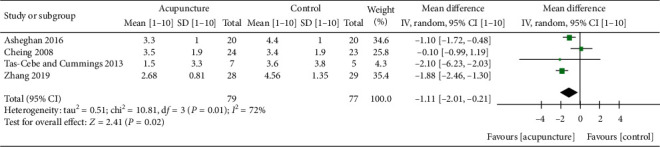
Forest plot of VAS score of EA and MA at 1- to 3-month follow-up. Forest plot of comparison: acupuncture (EA + PT exercise, MA + PT, MA + ESWT) versus control (PT + ESWT, PT exercise, IFE + PT exercise). Outcome: VAS score [1–10]. Follow-up time: [26] 1 month, [23] 1.5 months, [19] 2 months, and [22] 3 months. ^*∗*^Sensitivity analysis found that when excluding [19] from the analysis, the heterogeneity: tau^2^ = 0.12; chi^2^ = 3.34, d*f* = 2 (*P*=0.19); *I*^2^ = 40%, test for overall effect: *Z* = 1.36 (*P*=0.17).

One of the most common symptoms of FS is pain. The most popular way to measure pain is by the VAS. A meta-analysis was conducted with four studies and showed significant pain reduction in favor of MA versus the control (PT, sham acupuncture) at 0- to 12-week follow-up time (MD: −1.47; 95% CI: −1.87, −1.07; *P* < 0.00001; *I*^2^ = 25%) (Figure 3(a)) [19, 21–23]. At 4- to 6-week follow-up time, a meta-analysis of three studies also indicated positive tendencies reducing pain with the use of MA compared to the control (PT + ESWT, PT exercise) although with high heterogeneity (MD: −1.36; 95% CI: −2.3, −0.43; *P*=0.004; *I*^2^ = 60%) (Figure 3(b)) [19, 22, 23]. At 1.5- to 3-month follow-up time, a meta-analysis of three studies also showed significant pain reduction in favor of MA versus control (PT + ESWT, PT exercise) (MD: −1.52; 95% CI: −1.94, −1.11; *P* < 0.00001; *I*^2^ = 40%) (Figure 3(c)) [19, 22, 23]. Those analyses suggest that MA can be useful for pain reduction in the short and midterm. Additionally, a meta-analysis of both EA and MA compared to the control (PT + ESWT, PT exercise, IFE + PT exercise) at 1- to 3-month follow-up suggested a greater pain reduction in EA and MA compared to the control, although with high heterogeneity between studies (MD: −1.11; 95% CI: −2.01, −0.21; *P*=0.02; *I*^2^ = 72%) (Figure 4) [19, 22, 23, 26]. Initially, we considered two more studies in the meta-analysis [18, 27]. An unsuccessful attempt was made to contact Ma et al. and Lo et al. for missing standard deviation data, and as a result, we could not include these studies in the meta-analysis [18, 27].

When it comes to MA, seven studies found a significant reduction in VAS score. The follow-up time of studies varies from 10 days to 1 year [19–23, 25, 27]. Schroder et al. also showed an immediate reduction of pain in CMS pain subscore using distal acupuncture in a sham control double-blind trial [21] (Table 2). In regard to EA studies, a good improvement in VAS score was found in EA with the following point combination: Jian Qian (extra point), Jian Yu (LI15), Jian Liao (TB14), Nao Shu (SI10), Wai Guan (TB5), and He Gu (LI4) that reduced the mean VAS score from 6.4 before treatment to 2.5 after 10 days. The treatment consists of 5 treatments in 10 days [25]. Another study had a good reduction in VAS pain score with EA from 6.5 before treatment to 3.1 at four weeks and to 1.7 at six months, although the competitive treatment interferential electrotherapy (IFE) had similar significant pain reduction [25, 26].

Four studies use a total efficiency score (patients with posttreatment symptoms were divided into 3 groups: cured, with no pain sensation reported and restored ROM; improved, with reduced pain sensation and improvement in ROM; and failed, with no reported alleviation of pain sensation and no improvement in ROM), and the studies also showed a significant improvement in FS using acupuncture [20, 24, 25, 29]. The studies compared different types of acupuncture and acupoint stimulation methods. A study that includes 272 participants compared EA on mainly local points Jian Yu (LI15), Bi Nao (LI14), Jian Liao (TB14), Jian Zhen (SI9), Tian Zong (SI11), and Jian Jing (GB21) versus MA on two distal points San Jian (LI3) and Ling Xia (extra point); 20 treatments in 22 days were given in each group [29]. The results showed a significant improvement in the MA group with 158 (out of 210) cases of the acupuncture group which were marked as cured after the end of the treatment.

### 3.3. Shoulder Function

**Figure 5:**
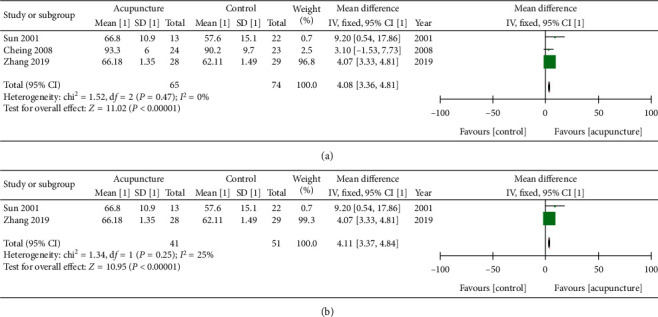
(a) Forest plot of shoulder function CMS score of EA and MA at 1.5–3 months. Forest plot of comparison: acupuncture (MA + exercise, EA + exercise, MA ESWT) versus control (IFE + PT exercise, PT exercise, PT exercise + ESWT). Follow-up time: [30] 1.5 months, [26] 3 months, and [19] 2 months. (b) Forest plot of shoulder function CMS score of MA at 1.5–2 months. Forest plot of comparison: acupuncture (MA + PT exercise, MA + ESWT) versus control (PT exercise, PT exercise + ESWT). Follow-up time: [30] 1.5 months and [19] 2 months. Outcome: CMS shoulder function score [0–100].

In regard to shoulder function, a meta-analysis using the CMS shoulder function score that included three studies and compared EA and MA versus control (IFE + PT exercise, PT exercise, PT exercise + ESWT) at a 1.5- to 3-month follow-up showed an improvement in shoulder function with the use of EA and MA compared to the control (MD: 4.08; 95% CI: 3.36, 4.81; *P* < 0.00001; *I*^2^ = 0%) (Figure 5(a)) [19, 26, 30]. A meta-analysis of two studies comparing MA with the control (PT exercise, PT exercise + ESWT) also showed significant (MD: 4.11; 95% CI: 3.37, 4.84; *P* < 0.00001; *I*^2^ = 25%) (Figure 5(b)) [19, 30]. The different shoulder function measurement tools across studies made it difficult to perform a larger meta-analysis. However, we analyzed shoulder function data in individual studies in Table 3. Seven studies used different methods to measure shoulder function and showed a significant improvement in shoulder function in the acupuncture groups [19, 20, 23–26, 30]. Four studies used the CMS score [19, 20, 26, 30]. Two studies used SPADI [18, 22], and one study used the OSS [23]. Two studies used less common measurement tools that the calculation methods are not fully described [24, 25]. EA exhibited a larger significant improvement in shoulder function compared to MA [25]. Acupuncture with shoulder exercise was more significant than shoulder exercise alone [22, 23, 26, 30].

### 3.4. Range of Motion (ROM)

**Figure 6:**
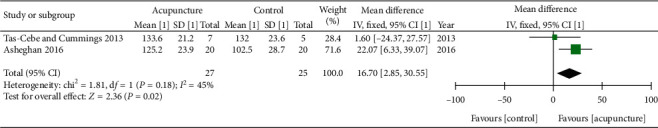
Forest plot of active flexion ROM score at 1.5-month follow-up. Forest plot of comparison: acupuncture (MA with PT exercises) versus control (PT exercises). Outcome: active flexion ROM [0–180].

**Figure 7:**
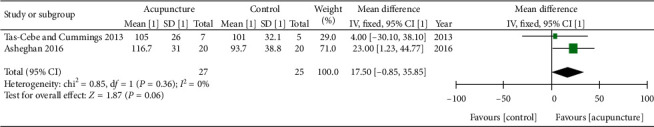
Forest plot of active abduction ROM at 1.5-month follow-up. Forest plot of comparison: acupuncture (MA with PT exercises) versus control (PT exercises). Outcome: active abduction ROM [0–180].

**Figure 8:**
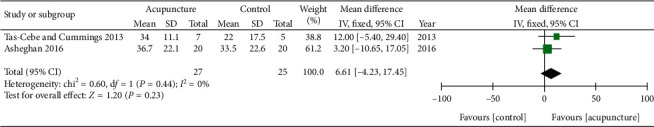
Forest plot of active external rotation ROM at 1.5-month follow-up. Forest plot of comparison: acupuncture (MA with PT exercises) versus control (PT exercises). Outcome: active external rotation ROM [0–180].

A meta-analysis for active flexion ROM of two studies comparing MA to PT at 6-week follow-up found significant in favor of MA (MD: 16.70; 95% CI: 2.85, 30.55; *P*=0.02; *I*^2^ = 45%) (Figure 6) [22, 23]. A meta-analysis for active ROM of abduction and external rotation including two studies [22, 23] failed to show significant results (MD: 17.50; 95% CI: −0.85, 35.85; *P*=0.06, *I*^2^ = 0% and MD: 6.61; 95% CI: −4.23, 17.45; *P*=0.23; *I*^2^ = 0%, respectively) (Figures 7 and 8). Initially, we planned to include two additional studies in the meta-analysis [27, 28], but we could not include Lin et al. [28] and Ma et al. [27] studies in the meta-analysis due to short follow-up (immediate effect in Lin et al. study) and missing standard deviation values in Ma et al. study. We analyzed shoulder ROM data in Table 4. Three individual studies investigate the effect on ROM, and acupuncture showed a significant improvement in ROM over time [22, 23, 27]; nevertheless, acupuncture improvement in ROM failed to outperform PT interventions in two of the studies [23, 27]. One study used video analysis that examined the effect of flexion ROM using EA at Tiao Kou (ST38), compared with EA at two sham points. Immediately after the treatment, there was an improvement in 8 degrees in the flexion ROM in the verum EA group (Table 4) [28].

The most used local points in the included studies are Jian Yu (LI15) that was used in eight studies, Jian Liao (TB14) that was used in six studies, and Bi Nao (LI14) and Nao Shu (SI10) that were used in three studies. In terms of distal points, He Gu (LI4) was used in five studies, Tiao Kou (ST38) was used in four studies, and Wai Guan (TB5) was used in two studies (Supplementary Table 5). The most common meridians used are large intestine—nine studies (69.2%), triple burner—eight studies (61.5%), small intestine—seven studies (53.8%), gallbladder—three studies (23%), stomach—four studies (30%), and bladder—two studies (15.3%).

### 3.5. Frozen Shoulder Stage

In our review, the included studies treated all stages of FS, two studies treated patients in the freezing stage [24, 25] and eight studies treated patients in the frozen stage which is considered as the most difficult to treat [19, 20, 22, 23, 26, 27, 29, 30], and one study included patients in the thawing stage [21]. Two studies failed to report the FS stage of treated patients [18, 28].

### 3.6. Adverse Events

No serious adverse events were reported in the included studies. Three studies reported that they did not have any adverse events [18, 21, 23]. Ten studies did not include an adverse events section [19, 20, 22, 24–30].

### 3.7. Strength of Evidence (GRADE)

There is very low evidence that acupuncture has a superior effect on FS when comparing to control interventions (Supplementary Table 6).

## 4. Discussion

FS is a condition that tremendously affects one's quality of life. The lack of a successful understanding of the causes and the lack of successful treatment for this condition leave patients to struggle with this long and painful condition and call for more research on alternative treatment methods. The goal of this review and meta-analysis is to determine the effects of acupuncture and EA on FS in terms of pain reduction and restoring ROM and improving shoulder function.

This review and meta-analysis include thirteen control studies on different methods of acupuncture including MA and EA for the treatment of FS. The studies compared acupuncture versus sham acupuncture, PT, home exercises, corticosteroid injection, IFE, and ESWT. In this meta-analysis, we found that MA and EA can be successful treatments for FS in regard to pain reduction, restoring shoulder function, and active flexion ROM (Figures 3–6). For shoulder function, seven studies showed significant results in favor of acupuncture. However, most of the studies measured shoulder function using different tests [19, 20, 23–26, 30]. In terms of restoring external rotation and abduction ROM, acupuncture did not show a significant improvement versus other interventions.

The majority of the patients in this review were in the frozen phase of FS. The effect of different acupuncture methods on pain reduction measured by VAS score was significant in seven studies [19, 20, 22, 23, 25–27]. In a previous meta-analysis, acupuncture was a useful treatment for musculoskeletal pain with the effect lasting at least one year [31]. An animal study that induced FS to rabbits found that acupuncture can reduce pain-producing factors, inflammation, and DNA expression to alleviate adhesions [32].

Poor blood circulation in the shoulder joint and lack of fluids in the shoulder bursa lead to inflammation and pain. The use of EA allows a consistent stimulation of the acupoints which leads to a stronger circulation of blood in the shoulder area, improvement in nourishment, and reduction in pain sensation. A study on gout arthritis in rats may help to explain the analgesic effect of EA by activating *μ* and *κ* opioid receptors [33]. This study also found that EA can significantly increase the expression of *β*-endorphin in local arthritis tissue. EA probably holds the ability to increase the body endogenous opioid system which can reduce the pain sensation. EA was also mentioned in Jain & Sharma's review article as a useful treatment for pain management for FS [2].

Distal acupuncture also showed an immediate increase in the ROM and immediate reduction of pain in VAS score [19–21, 28]. A recent systematic review and meta-analysis on the effect of acupuncture at Tiao Kou (ST38) for FS also found significant improvement in clinical effectiveness and can also support the concept of distal acupuncture, although the mechanism of distal acupuncture is still unknown [34]. One study includes moxibustion on top of the acupuncture needles [25]. The study involved 3 groups: MA, EA, and moxibustion. In regard to pain reduction, there was a significant improvement in all 3 groups and no significant difference between the groups. All 3 groups improved significantly in ROM as well, when the moxibustion group had a slightly higher degree of ROM improvement than the other 2 groups, although not a significant difference. Another study interestingly suggests that proper distal point selection in MA might have a greater effect when compared to EA [29]. This highlights the complicity in acupuncture interventions where the acupuncturist can choose from hundreds of potential acupoints. A recent meta-analysis on acupotomy for FS found that acupotomy is efficient and safe therapy and can generate a significantly greater pain reduction when compared to acupuncture [35]. A review of fire needles from Korea that was published in 2013 focused on Chinese studies and included 23 studies. 22 studies reported a significant improvement in fire needling on frozen shoulder patients. Out of the 22 studies, 7 control studies showed that fire needling achieved better results than MA and EA [36]. The effect can be explained by the fact that the big amount of frozen shoulder causes was described in Chinese medicine to be caused by cold or dampness bi syndrome. Both coldness and dampness can be eliminated by the direct heat generated by fire needling or moxibustion. In regard to improving shoulder function, although most studies measured shoulder function using different tests, a fact that made it difficult to conduct a meta-analysis, seven individual studies showed significant results in favor of acupuncture [19, 20, 23–26, 30]. Acupuncture can also improve mental conditions like sadness and improve sleep quality which is essential for the healing process and may improve ADL [37].

The most common points used to treat FS in the review are Jian Yu (LI15) (8 studies) and Jian Liao (TB14) (6 studies). Those points are located in key positions around the shoulder capsule, Jian Yu (LI15) is located in the anterosuperior joint capsule and in close proximity to the coracohumeral ligament, and the Jian Liao (TB14) is located in the rotator cuff interval areas that are reported as inflamed area that develops adhesions in FS patients. Those local points can improve blood circulation in the shoulder and reduce pain. The results of this systematic review and meta-analysis suggest that both EA and MA in local and distal acupoints can activate the acupoint therapeutic ability and generate this beneficial effect. Peng et al. in 2007 conducted a previous review and meta-analysis on acupuncture and frozen shoulder [10]. The review concluded that acupuncture is a safe treatment. Limited evidence showed that acupuncture can be effective in regard to pain reduction, improving ROM, and shoulder function. Peng's review only included 6 studies and did not exclude rotator cuff disease and osteoarthritis. Our review includes 13 studies and excluded rotator cuff disease and osteoarthritis patients. Our review also includes an updated quality assessment tool like the “STRICTA 2010” checklist and the Cochrane Collaboration's risk of bias tool.

## 5. Limitations and Problems

In all meta-analysis, the strength of evidence (GRADE) was very low (Supplementary Table 6). Some of the studies failed to provide a reliable measurement method, adequate patient randomization, and allocation. All of the studies have an unknown publication bias and we could not find any published study protocols for the included studies. In this review, we made two unsuccessful attempts to contact Ma et al. and Lo et al. studies for missing data. Most of the studies investigating FS are in the Chinese language which stands as an obstacle for this review. Only 3 studies out of the 13 studies included compared the acupuncture group to a sham acupuncture group in order to eliminate the placebo effect [18, 21, 28]. The length of most of the studies' follow-up is around 1–3 months, and a longer follow-up in this condition is needed. The follow-up time of 1 year should stand as the standard follow-up for this condition, due to the long recovery time without treatment of 1–4 years [4]. The large variety of measurement tools in FS studies made conducting a larger meta-analysis impossible. We recommend future acupuncture studies to measure pain using the VAS. For shoulder function measurements, we recommend acupuncture studies to focus on reliable tools such as SPADI, Disabilities of the Arm, Shoulder, and Hand (DASH) questionnaire, or the American Shoulder and Elbow Surgeons (ASES) [38]. Future studies should investigate the effect of acupuncture compared to injected steroids, arthroscopic capsular release, hydrodilatation, manipulation under anesthesia, and low-level laser.

## 6. Conclusions

In this systematic review and a meta-analysis, acupuncture had shown to be a safe treatment with a significant effect in regard to reducing pain, improving shoulder function, and flexion ROM in the short term and midterm. However, due to the small number of included studies and methodological limitations in these studies, more large-scale high-quality RCTs are warranted in order to give a robust conclusion. Future studies should compare acupuncture to other treatments and sham acupuncture. Additionally, longer follow-up time is needed for investigating the effect of acupuncture in the mid- and long term, and the duration of future FS studies follow-up should be increased to one year.

## Figures and Tables

**Table 1:** Main findings and characteristics of studies included in the systematic review.

Study	Cases	Intervention	FS duration (mean)	Sessions/weeks	Outcome measurement	Results

[18]	21	(1) EA + PT (*n* = 11): LI15, TE14, SI10, GB34, ST38 (2) Sham EA + PT (*n* = 10): sham acupoints 1-2 cm around the actual points	Did not describe. Inclusion criteria less than 3 months	18 sessions 2-3 sessions per week. Follow-up post 18 sessions and 1, 3, and 6 months post 18 sessions.	VAS, ROM, SPADI	A significant decrease in VAS and an increase in SPADI, ROM between the baseline and the follow-up periods, no significant change between the two groups in VAS, SPADI. ROM in the follow-up periods.

[19]	57	(1) MA + PT exercise + ESWT (*n* = 28): acupoint in the middle of the forehead (between GV24 and Yintang), BL1, LU10, LI4, LI15, TB3, SI3, SI10 (2) PT exercise + ESWT (*n* = 29)	4.13 ± 1.96 months in the MA group and 4.70 ± 2.26 months in the control group	PT exercise, MA, ESWT, 6 times a week for 4 weeks Follow-up: after the treatment course, telephone calls 30 days after the treatment course.	VAS, CMS, HAMA, cured, effective, or ineffective	Significant improvement in both groups in all measurements from baseline. MA group had a significant reduction in VAS and in HAMA compared to the control group at both follow-up periods. CMS was significantly increased in the MA group compared to the control in both follow-up periods.

[20]	64	(1) MA-Ashi points (*n* = 32): LI4, LI20, TE3, TE23, SI3, SI19 (2) Ashi points (*n* = 32)	4.56 ± 1.25 months in the treatment group and 4.84 ± 2.62 in the control group	Both groups received the interventions once a day, 6 times per week. The total treatment time was 4 weeks. Follow-up at 1 and 2 months.	VAS, CMS, HAMA, cured, effective, or ineffective	Significant improvement of both groups posttreatment and in the follow-up compared to baseline in all measurements. The treatment group has significantly improved in all measurements and in both follow-up times compared to the control.

[21]	60	(1) A double-blind (2) ACU with press tack needles (*n* = 30): using reflex areas in the distant extremities (3) Press tack placebos (*n* = 30): same method with placebo needles	ACU 16.0 ± 23.6 months Placebo 15.6 ± 18.8 months	Participants received just one treatment Effect measured immediately after treatment	CMS pain subscore	Improvement of 3.3 points in ACU vs. 1.6 points in the placebo group (*P* < 0.02).
Follow-up study [21]	47	(1) MA + conservative therapy (NSAID) (*n* = 34) (2) Conservative therapy only, (*n* = 13) (treatment includes NSAIDs, steroid injections, oral opioid, TENS)	16 months (47 of the 60 proceed to the follow-up study)	MA 10 treatments in 10 weeks Conservative therapy for up to 1 year 1 year follow-up	CMS pain subscore	After 1 year increase of CMS pain subscore of 5.7 ± 3.8 points in the MA group and 5.1 ± 4.5 points in the conservative therapy group. MA group reaches the final goal significantly faster (*P* < 0.001).

[22]	40	(1) MA + PT (*n* = 20) acupoints did not describe (2) PT (*n* = 20)	MA + PT group 4.05 ± 2.06 months and control group 4.10 ± 2.17 months	MA: twice a week for 20 treatments (1.5 months) PT: each the other day until the last session of MA for all groups (1.5 months)	SPADI, VAS, ROM Follow-up: 1.5 months 3 months	In ROM, MA + PT was higher than the control (*P* < 0.05), after 3 months, the VAS reduced significantly in the MA + PT comparing to the control group (*P* < 0.05). No difference in SPADI between the 2 groups.

[23]	20	(1) Home exercises (*n* = 5) (2) Corticosteroid injection with home exercises (*n* = 8) (3) MA with home exercises (*n* = 7): LI15, SJ14, LI14, SI11, SI12, SI14, LI4, ST38	7 months	Exercise twice a day for 6 weeks (all groups). MA once a week for 6 weeks Measurements after treatments.	OSS, ROM, VAS scores	At 6 weeks, improvements in pain, function, and ROM in all groups. No significant difference between the groups.

[24]	64	(1) MA + AQF (*n* = 32) (2) MA (*n* = 32) acupoints: LI15, TE14, SI9, LI 14, LI11, TE5 in both groups	MA + AQF 3.46 ± 1.57 months MA 3.42 ± 1.48 months	Treatment once daily for 12 days Effect measured after treatment	Cured, improved, or failed ADL	Statistically significant improvement in both groups (*P* < 0.05). The MA + AQF group was better than the MA group in all aspects.

[25]	174	(1) MA group (*n* = 56) (2) EA group (*n* = 57) (3) MOXA group (*n* = 61) acupoints: Jianqian, LI15, TE14, SI10, TE5 (EA), LI4 (EA)	2.97 ± 0.33 months	5 treatments in total 10 days After 10 days, the effect was evaluated.	VAS, ROM, cured, improved, or failed	Significant improvement in all 3 groups in ROM and analgesic effect. EA made more analgesic effects and MOXA made more improvement in the shoulder ROM.

[26]	70	(1) EA + exercise (*n* = 24): LI15, 1 trigger point (EA), ST38 (MA) (2) IFE + exercise (*n* = 23) (3) Control group (*n* = 23): no treatment	EA: 6.71 ± 6.5 months IFE: 6.70 ± 6.05 months control: 8.26 ± 7.94 months	10 sessions over a 4-week period (2-3 times a week) Effect measured after treatments and at 6 months	VAS, CMS	In EA and IFE groups, the increased CMS and VAS decreased significantly (both *P* < 0.001). No significant in the control group and no significant difference between the 2 intervention groups.

[27]	75	(1) MA only (*n* = 30): TB14, LI15, GB20, LI4, GB34 (2) PT: control (*n* = 30) (3) MA + PT (*n* = 15)	Mean 25.8 weeks in all 3 groups	MA: 4 weeks, 8 treatments PT: 4 weeks, 20 treatments. The effect was measured at 2 and 4 weeks.	ROM, VAS, SF-36 Health Survey (ADL)	All groups improved in quality of life. The pain was reduced more by MA while ROM improved more in PT. MA + PT had the best outcome.

[28]	14	(1) EA: ST38 (*n* = 14) (2) Sham: 2 sham acupoints + EA(*n* = 14) (all of the patients received both true and sham ACU)	Did not describe	3 times for treatment Measurement before and after the interventions	Video-based humeral elevation	Humeral elevation ROM increased in 8.34 degrees in the true ACU group, significantly better than in the sham group.
[29]	272	(1) MA + exercise (*n* = 210): LI3 and Lingxia (2) Control (*n* = 62): EA—LI15, LI14, TB14, SI9, SI11, GB21	Mean 4.6 months (in both groups)	20 treatments in 22 days. The effect was measured after treatment.	Cured, improved, or failed	MA + exercise: 158 cases were cured, 40 cases improved, and 12 cases failed. Control: 26 cases were cured, 21 improved, and 15 failed. MA + exercise compared to control (*P* < 0.01).

[30]	35	(1) PT exercise (*n* = 22) (2) MA + PT exercise (*n* = 13): Zhongping extra point Contralateral deep needling	7.1 ± 3.9 months in the PT group and 5.5 ± 1.6 months in the PT + MA group	6 weeks twice a week. The effect was measured after treatment and after 20 weeks.	CMS	Significantly higher CMS in the PT exercise + MA group compared with the PT exercise group at 20 weeks (*P*=0.048).

ACU: acupuncture; ADL: ability in daily life; AQF: accelerating qi-flow along meridians; CMS: Constant-Murley Score; EA: electroacupuncture; IFE: interferential electrotherapy; MA: manual acupuncture; MOXA: moxibustion; OSS: Oxford Shoulder Score (scoring system of 12–60, with 12 being the best outcome); PT: physical therapy; ROM: range of motion; TCM: traditional Chinese medicine; TENS: transcutaneous electrical nerve stimulation; ESWT: extracorporeal shockwave therapy; VAS: visual analogue scale; SPADI: Shoulder Pain and Disability Index; HAMA: Hamilton Anxiety Scale. Chinese name of acupoints: Ju Gu (LI16), Jian Yu (LI15), Bi Nao (LI14), Qu Chi (LI11), He Gu (LI4), San Jian (LI3), Jian Liao (TB14), Wai Guan (TB5), Jian Wai Shu (SI14), Bing Feng (SI12), Tian Zong (SI11), Nao Shu (SI10), Jian Zhen (SI9), Feng Chi (GB20), Jian Jing (GB21), Yang Ling Quan (GB34), Tiao Kou (ST38), Cheng Shan (BL57), Shen Ting (GV24), Jing Ming (BL1), Yu Ji (LU10), Zhong Zhu (TB3), Hou Xi (SI3), Ying Xiang (LI20), Sizhukong (TH23), and Ting Gong (SI19).

**Table 2:** VAS scores for acupuncture treatments of frozen shoulder.

Study	Treatment group	Baseline	1 week	10 days	4 weeks	6 weeks	8 weeks	12 weeks	6 months	1 year

[27]	PT	1.8			0.4					
	Acupuncture	2.3			0.7					
	PT + acupuncture	2.6			0.7					

[26]	Exercise + EA	6.5 ± 2.1			3.1 ± 2.2			2.4 ± 2.2	1.7 ± 2.3	
	Exercise + IFE	6.5 ± 2.0			2.4 ± 1.7			2.0 ± 1.5	1.3 ± 1.4	

[25]	Acupuncture	6.44 ± 0.15		3.67 ± 0.23						
	EA	6.80 ± 0.13		2.52 ± 0.24						
	Moxibustion	6.70 ± 0.16		2.96 ± 0.19						

[23]	Exercise	3.8 ± 3.8				3.6 ± 3.8				
	Exercise + steroid injection	3.7 ± 2.7				1.6 ± 2.2				
	Exercise + acupuncture	3.9 ± 3.8				1.5 ± 3.3				

[22]	PT + acupuncture	8 ± 1				5 ± 1		3.3 ± 1		
	PT	7.9 ± 1				5.8 ± 1		4.4 ± 1		

[19]	Exercise + ESWT + acupuncture	6.67 ± 1.43			3.47 ± 1.35		2.68 ± 0.81			
	Exercise + ESWT	7.57 ± 1.31			5.36 ± 1.45		4.56 ± 1.35			

[20]	MA-Ashi + distal points	5.65 ± 1.59			3.16 ± 1.67		2.43 ± 0.98			
	MA-Ashi points	6.03 ± 0.96			4.89 ± 2.23		3.79 ± 1.45			

EA: electroacupuncture; MA: manual acupuncture; PT: physical therapy; ESWT: extracorporeal shockwave therapy.

**Table 3:** Shoulder function scores from acupuncture studies.

Assessment tool	Study	Outcome

ADL	[24] AQF	Baseline 1.56 ± 0.51; at 12 days 4.16 ± 0.67
	[24] ACU	Baseline 1.53 ± 0.46; at 12 days 3.02 ± 0.63

CMS	[30] Exercise	Baseline 42.8 ± 14.0; at 6 weeks: 57.6 ± 15.1; at 5 months 57.9 ± 15.1
	[30] Exercise + MA	Baseline 41.3 ± 14.9; at 6 weeks: 66.8 ± 10.9; at 5 months 67.3 ± 11.5
	[26] Exercise + EA	Baseline 65.5 ± 16.7; at 1 month 89.3 ± 4.8; at 3 months 93.3 ± 6.0; at 6 months 93.8 ± 6.4
	[26] Exercise + IFE	Baseline 59.6 ± 15.4; at 1 month 92.1 ± 5.9; at 3 months 90.2 ± 9.7; at 6 months 95.5 ± 4.1
	[19] Exercise + ESWT + acupuncture	Baseline 54.65 ± 1.65; at 1 month 64.54 ± 2.19; at 2 months 66.18 ± 1.35
	[19] Exercise + ESWT	Baseline 54.32 ± 2.31; at 1 month 61.01 ± 0.95; at 2 months 62.11 ± 1.49
	[20] MA-Ashi + distal points	Baseline 56.98 ± 1.71; at 1 month 66.35 ± 1.39; at 2 months 62.28 ± 1.45
	[20] MA-Ashi points	Baseline 55.92 ± 2.35; at 1 month 62.28 ± 1.45; at 2 months 64.03 ± 0.93

OSS	[23] Exercise only	Baseline 33.6 ± 6.1; at 6 weeks: 32.6 ± 5.0
	[23] Exercise + corticosteroid injection	Baseline 38.5 ± 9.0; at 6 weeks: 28.3 ± 11.1
	[23] MA + exercises	Baseline 37.3 ± 9.3; at 6 weeks: 24.3 ± 7.4

Shoulder activity degree	[25] MA	Baseline 237.3 ± 8.14; at 10 days: 279.8 ± 6.6
	[25] EA	Baseline 238.6 ± 7.3; at 10 days: 299 ± 6.5
	[25] Moxibustion	Baseline 227.5 ± 8.6; at 10 days: 304.4 ± 6.0

SPADI	[22] MA	Baseline 87.9 ± 15; at 1.5 months 62.1 ± 14; at 3 months 41.9 ± 16
	[22] PT	Baseline 88.8 ± 14; at 1.5 months 67.8 ± 9; at 3 months 49.7 ± 13

ADL: ability in daily life; AQF: accelerating qi-flow along meridians; CMS: Constant-Murley Score; EA: electroacupuncture; IFE: interferential electrotherapy; MA: manual acupuncture; OSS: Oxford Shoulder Score (scoring system of 12–60, with 12 being the best outcome); PT: physical therapy; SPADI: Shoulder Pain and Disability Index.

**Table 4:** Active ROM scores for acupuncture treatments of frozen shoulder.

Study	Treatment	Flexion	Abduction	External rotation

[28]	EA	Baseline 102.91 ± 24.82 to 111.25 ± 22 immediately after		
	Sham EA	Baseline 102.91 ± 24.82 to 103.90 ± 22.79 immediately after		

[27]	MA	Baseline 109.1; at 4 weeks 125.0	Baseline 88.8; at 4 weeks 90.7	Baseline 32.1; at 4 weeks 42.7
	MA + PT	Baseline 106.5; at 4 weeks 130.2	Baseline 79.5; at 4 weeks 90.3	Baseline 20.7; at 4 weeks 30.1
	PT	Baseline 107.7; at 4 weeks 124.4	Baseline 70.6; at 4 weeks 84.9	Baseline 15.9; at 4 weeks 27.3

[23]	Exercise only	Baseline 106.0 ± 15.1; at 6 weeks 132.0 ± 23.6	Baseline 84.0 ± 26.1; at 6 weeks 101.0 ± 32.1	Baseline 10.0 ± 12.3; at 6 weeks 22.0 ± 17.5
	Exercise + steroid injection	Baseline 98.8 ± 27.0; at 6 weeks 125.0 ± 33.0	Baseline 72.5 ± 33.30; at 6 weeks 95.0 ± 36.6	Baseline 16.3 ± 25; at 6 weeks 35.0 ± 26.9
	Exercise + MA	Baseline 120.0 ± 16.6; at 6 weeks 133.6 ± 21.2	Baseline 89.3 ± 18.6; at 6 weeks 105.0 ± 26.0	Baseline 30.0 ± 11.5; at 6 weeks 34.0 ± 11.1

[22]	MA + PT	Baseline 83.5 ± 35.2; at 1.5 months 125.2 ± 23.9; at 3 months 149 ± 19.09	Baseline 72.7 ± 40.8; at 1.5 months 116.7 ± 31; at 3 months 146 ± 34.7	Baseline 19.8 ± 24.2; at 1.5 months 36.7 ± 22.1; at 3 months 49 ± 19.8
	PT	Baseline 81.7 ± 37.6; at 1.5 months 102.5 ± 28.7; at 3 months 125.2 ± 21.3	Baseline 73.7 ± 41.3; at 1.5 months 93.7 ± 38.8; at 3 months 112.5 ± 32.4	Baseline 23.8 ± 25.3; at 1.5 months 33.5 ± 22.6; at 3 months 40.25 ± 22.1

EA: electroacupuncture; MA: manual acupuncture; PT: physical therapy.

## Data Availability

This manuscript is a review of published studies when all the data are publicly available.

## Supplementary Materials

Supplementary Materials Supplementary Table 1: PRISMA checklist. Supplementary Table 2: frozen shoulder papers search from databases. Supplementary Table 3: GRADE score for included studies. Supplementary Table 4: checklist for items in “STRICTA 2010” for acupuncture reporting. Supplementary Table 5: acupoints used in included studies. Supplementary Table 6: GRADE recommendations for the level of certainty in a meta-analysis.
