# Supplementary material for: The Effectiveness of Acupuncture in the Treatment of Frozen Shoulder: A Systematic Review and Meta-Analysis
Source: Evid Based Complement Alternat Med. 2020 Sep 25;2020:9790470. doi: 10.1155/2020/9790470 (PMC7532995; doi:10.1155/2020/9790470)
Supplement: Supplementary Materials — Supplementary Table 1: PRISMA checklist. Supplementary Table 2: frozen shoulder papers search from databases. Supplementary Table 3: GRADE score for included studies. Supplementary Table 4: checklist for items in “STRICTA 2010” for acupuncture reporting. Supplementary Table 5: acupoints used in included studies. Supplementary Table 6: GRADE recommendations for the level of certainty in a meta-analysis. [file 9790470.f1.docx]

**Supplementary Table 1.** PRISMA checklist filled up for this review.

| **Section/topic** | **#** | **Checklist item** | **Reported on page #** |
| --- | --- | --- | --- |
| **TITLE** | | |  |
| Title | 1 | Identify the report as a systematic review, meta-analysis, or both. | 1 |
| **ABSTRACT** | | |  |
| Structured summary | 2 | Provide a structured summary including, as applicable: background; objectives; data sources; study eligibility criteria, participants, and interventions; study appraisal and synthesis methods; results; limitations; conclusions and implications of key findings; systematic review registration number. | 2 |
| **INTRODUCTION** | | |  |
| Rationale | 3 | Describe the rationale for the review in the context of what is already known. | 3 |
| Objectives | 4 | Provide an explicit statement of questions being addressed with reference to participants, interventions, comparisons, outcomes, and study design (PICOS). | 3 |
| **METHODS** | | |  |
| Protocol and registration | 5 | Indicate if a review protocol exists, if and where it can be accessed (e.g., Web address), and, if available, provide registration information including registration number. | 4 |
| Eligibility criteria | 6 | Specify study characteristics (e.g., PICOS, length of follow-up) and report characteristics (e.g., years considered, language, publication status) used as criteria for eligibility, giving rationale. | 4 |
| Information sources | 7 | Describe all information sources (e.g., databases with dates of coverage, contact with study authors to identify additional studies) in the search and date last searched. | 4 |
| Search | 8 | Present full electronic search strategy for at least one database, including any limits used, such that it could be repeated. | 4 |
| Study selection | 9 | State the process for selecting studies (i.e., screening, eligibility, included in systematic review, and, if applicable, included in the meta-analysis). | 4, Figure 1 |
| Data collection process | 10 | Describe method of data extraction from reports (e.g., piloted forms, independently, in duplicate) and any processes for obtaining and confirming data from investigators. | 4 |
| Data items | 11 | List and define all variables for which data were sought (e.g., PICOS, funding sources) and any assumptions and simplifications made. | 4 |
| Risk of bias in individual studies | 12 | Describe methods used for assessing risk of bias of individual studies (including specification of whether this was done at the study or outcome level), and how this information is to be used in any data synthesis. | 5-6 |
| Summary measures | 13 | State the principal summary measures (e.g., risk ratio, difference in means). | 6 |
| Synthesis of results | 14 | Describe the methods of handling data and combining results of studies, if done, including measures of consistency (e.g., I^2^) for each meta-analysis. | 6 |
| Risk of bias across studies | 15 | Specify any assessment of risk of bias that may affect the cumulative evidence (e.g., publication bias, selective reporting within studies). | 5, Supplementary Table 6 |
| Additional analyses | 16 | Describe methods of additional analyses (e.g., sensitivity or subgroup analyses, meta-regression), if done, indicating which were pre-specified. | 6 |
| **RESULTS** | | |  |
| Study selection | 17 | Give numbers of studies screened, assessed for eligibility, and included in the review, with reasons for exclusions at each stage, ideally with a flow diagram. | 7, Figure 1 |
| Study characteristics | 18 | For each study, present characteristics for which data were extracted (e.g., study size, PICOS, follow-up period) and provide the citations. | Table 1 |
| Risk of bias within studies | 19 | Present data on risk of bias of each study and, if available, any outcome level assessment (see item 12). | 7-8, Figure 2 |
| Results of individual studies | 20 | For all outcomes considered (benefits or harms), present, for each study: (a) simple summary data for each intervention group (b) effect estimates and confidence intervals, ideally with a forest plot. | 8-10, Figures 3-8, Supplementary Figures 1-4 |
| Synthesis of results | 21 | Present results of each meta-analysis done, including confidence intervals and measures of consistency. | 8-10, Figures 3-8 |
| Risk of bias across studies | 22 | Present results of any assessment of risk of bias across studies (see Item 15). | Figure 2, Supplementary Table 6 |
| Additional analysis | 23 | Give results of additional analyses, if done (e.g., sensitivity or subgroup analyses, meta-regression [see Item 16]). | 8, Figure 4 |
| **DISCUSSION** | | |  |
| Summary of evidence | 24 | Summarize the main findings including the strength of evidence for each main outcome; consider their relevance to key groups (e.g., healthcare providers, users, and policy makers). | 11-12 |
| Limitations | 25 | Discuss limitations at study and outcome level (e.g., risk of bias), and at review-level (e.g., incomplete retrieval of identified research, reporting bias). | 13 |
| Conclusions | 26 | Provide a general interpretation of the results in the context of other evidence, and implications for future research. | 14 |
| **FUNDING** | | |  |
| Funding | 27 | Describe sources of funding for the systematic review and other support (e.g., supply of data); role of funders for the systematic review. | 14 |

From: Moher D, Liberati A, Tetzlaff J, Altman DG, The PRISMA Group (2009). Preferred Reporting Items for Systematic Reviews and Meta-Analyses: The PRISMA Statement. PLoS Med 6(7): e1000097. doi:10.1371/journal.pmed1000097.

**Supplementary Table 2.** Frozen shoulder papers search from databases through January 1999 to August 2020.

| **Keyword/Database** | **PubMed** | **Embase** | **Cochrane Library** | **Web of Science** |
| --- | --- | --- | --- | --- |
| acupuncture frozen shoulder | Studies: 13  Duplication:0  Included: 7 | Studies:77  Duplication:5  Included:2 | Studies:25  Duplication:12  Included: 1 | Studies: 28  Duplication:22  Included: 0 |
| acupuncture adhesive capsulitis | Studies: 11  Duplication:11  Included:0 | Studies: 32  Duplication:11  Included: 0 | Studies: 13  Duplication:10  Included: 0 | Studies:22  Duplication:21  Included: 0 |
| acupuncture periarthritis | Studies: 4  Duplication:4  Included: 0 | Studies: 121  Duplication:26  Included:3 | Studies: 77  Duplication:27  Included: 0 | Studies: 7  Duplication:6  Included:0 |
| electroacupuncture frozen shoulder | Studies: 2  Duplication:2  Included:0 | Studies: 12  Duplication:8  Included: 0 | Studies: 4  Duplication:4  Included: 0 | Studies: 5  Duplication:5  Included: 0 |
| electroacupuncture adhesive capsulitis | Studies: 1  Duplication:1  Included: 0 | Studies: 4  Duplication:4  Included: 0 | Studies:2  Duplication:2  Included:0 | Studies: 1  Duplication:1  Included: 0 |
| electroacupuncture periarthritis | Studies: 0  Duplication:0  Included: 0 | Studies: 12  Duplication:12  Included: 0 | Studies: 5  Duplication:5  Included: 0 | Studies: 0  Duplication:0  Included: 0 |
| **Total** | Studies: 31  Duplication:18  Included: 7 | Studies: 258  Duplication:66  Included: 5 | Studies: 126  Duplication:60  Included: 1 | Studies:63  Duplication:55  Included: 0 |

The table describes the databases and keywords used in the search of studies to include in this review “Included” means studies that were included in our review.

**Supplementary Table 3.** Grading of Recommendations Assessment, Development and Evaluation (GRADE) score for included studies.

| Study | Initial score | Lack of allocation concealment | Lack of blinding | Incomplete accounting of patients and outcome events | Selective outcome reporting | Other limitations | GRADE score |
| --- | --- | --- | --- | --- | --- | --- | --- |
| )Lo et al 2020([21] | High | ? | ? | + | ? | ? | Low |
| (Zhang et al. 2019)[18] | High | ? | - | + | + | ? | Moderate |
| (Zhang et al. 2019)[30] | High | ? | ? | + | + | + | Moderate |
| (Schroder et al. 2017)[29] | High | + | + | + | ? | + | High |
| )Ashegan et al. 2016([22] | High | ? | - | + | + | ? | Moderate |
| (Tas-Cebe and Cummings 2013)[25] | High | -1 | -1 | + | ? | + | Low |
| (Shang et al. 2012)[19] | High | ? | -1 | + | ? | -1 | Low |
| (Shi et al. 2012)[27] | High | ? | + | + | ? | + | Moderate |
| (Cheing et al. 2008)[20] | High | -1 | -1 | + | ? | + | Low |
| (Ma et al. 2006)[23] | High | -1 | -1 | + | ? | + | Low |
| (Lin et al. 2005)[28] | High | -1 | + | + | ? | -1 | Low |
| (Feng 2003)[26] | Low | -1 | -1 | + | -1 | -1 | Very low |
| (Sun et al. 2001)[24] | High | ? | ? | + | ? | + | Low |

Study GRADE reduction policy:

-1: the information is missing– study GRADE reduced 1 point.

? : the information wasn’t reported in a sufficient manner and two “?” marks in one study will reduce study GRADE 1 point.

+ : the information was reported well- study GRADE stays intact.

**Supplementary Table 4.** Checklist for items in “STRICTA 2010” for acupuncture reporting.

|  | 1a | 1b | 1c | 2a | 2b | 2c | 2d | 2e | 2f | 2g | 3a | 3b | 4a | 4b | 5 | 6a | 6b | total |
| --- | --- | --- | --- | --- | --- | --- | --- | --- | --- | --- | --- | --- | --- | --- | --- | --- | --- | --- |
| )Lo et al 2020([21] | 1 | 1 | 1 | 1 | 1 | 0 | 1 | 1 | 1 | 0 | 1 | 1 | 1 | 0 | 0 | 0 | 1 | 12 |
| (Zhang et al. 2019)[18] | 1 | 0 | 1 | 0 | 1 | 0 | 1 | 1 | 1 | 0 | 1 | 1 | 1 | 1 | 0 | 0 | 1 | 11 |
| (Zhang et al. 2019)[30] | 1 | 0 | 1 | 0 | 1 | 1 | 1 | 1 | 1 | 0 | 1 | 1 | 1 | 0 | 0 | 1 | 1 | 12 |
| (Schroder et al. 2017)[29] | 1 | 1 | 1 | 0 | 0 | 1 | 0 | 0 | 1 | 1 | 1 | 1 | 0 | 1 | 1 | 1 | 1 | 12 |
| )Ashegan et al. 2016([22] | 0 | 1 | 0 | 0 | 0 | 0 | 0 | 0 | 0 | 0 | 1 | 1 | 1 | 0 | 0 | 0 | 0 | 4 |
| (Tas-Cebe and Cummings 2013)[25] | 1 | 1 | 0 | 1 | 1 | 0 | 1 | 1 | 1 | 1 | 1 | 1 | 1 | 1 | 1 | 1 | 1 | 15 |
| (Shang et al. 2012)[19] | 1 | 1 | 1 | 1 | 1 | 0 | 1 | 1 | 1 | 1 | 1 | 1 | 0 | 1 | 0 | 0 | 1 | 13 |
| (Shi et al. 2012)[27] | 1 | 0 | 0 | 1 | 1 | 1 | 0 | 1 | 1 | 1 | 1 | 1 | 1 | 1 | 0 | 0 | 1 | 12 |
| (Cheing et al. 2008)[20] | 1 | 1 | 1 | 1 | 1 | 1 | 0 | 1 | 1 | 1 | 1 | 1 | 1 | 1 | 0 | 1 | 1 | 15 |
| (Ma et al. 2006)[23] | 1 | 1 | 0 | 1 | 1 | 0 | 0 | 0 | 1 | 0 | 1 | 1 | 1 | 0 | 0 | 1 | 1 | 10 |
| (Lin et al. 2005)[28] | 1 | 1 | 0 | 1 | 1 | 1 | 1 | 1 | 1 | 0 | 1 | 0 | 1 | 1 | 0 | 0 | 1 | 12 |
| (Feng 2003) [26] | 1 | 0 | 0 | 1 | 1 | 0 | 0 | 1 | 1 | 0 | 1 | 1 | 1 | 0 | 0 | 0 | 1 | 9 |
| (Sun et al. 2001)[24] | 1 | 1 | 0 | 1 | 1 | 1 | 1 | 1 | 1 | 1 | 1 | 1 | 1 | 1 | 1 | 1 | 1 | 16 |

Questions were scored yes = 1, no = 0. Highest score = 17 (good acupuncture methods reporting), lowest score = 0 (poor acupuncture methods reporting).

**Supplementary Table 5**. Acupoints used in studies.

| Study/Point | [29] | [21] | [18] | [30] | [22] | [19] | [26] | [28] | [25] | [27] | [24] | [23] | [20] |
| --- | --- | --- | --- | --- | --- | --- | --- | --- | --- | --- | --- | --- | --- |
| LI15 (Jian Yu( |  | V | V |  |  | V | V |  | V | V |  | V | V |
| LI14 (Bi Nao( |  |  |  |  |  | V | V |  | V |  |  |  |  |
| LI11 (Qu Chi( |  |  |  |  |  | V |  |  |  |  |  |  |  |
| LI4 (He Gu( |  |  | V | V |  |  |  |  | V | V |  | V |  |
| LI3 (San Jian( |  |  |  |  |  |  | V |  |  |  |  |  |  |
| LU10 (Yu Ji) |  |  | V |  |  |  |  |  |  |  |  |  |  |
| TB14 (Jian Liao ( |  | V |  |  |  | V | V |  | V | V |  | V |  |
| TB5 (Wai Guan ( |  |  |  |  |  | V |  |  |  | V |  |  |  |
| SI14 (Jian Wai Shu) |  |  |  |  |  |  |  |  | V |  |  |  |  |
| SI12 (Bing Feng ( |  |  |  |  |  |  |  |  | V |  |  |  |  |
| SI11 (Tian Zong ( |  |  |  |  |  |  | V |  | V |  |  |  |  |
| SI10 (Nao Shu ( |  | V | V |  |  |  |  |  |  | V |  |  |  |
| SI9 (Jian Zhen ( |  |  |  |  |  | V | V |  |  |  |  |  |  |
| GB20 (Feng Chi ( |  |  |  |  |  |  |  |  |  |  |  | V |  |
| GB21 (Jian Jing ( |  |  |  |  |  |  | V |  |  |  |  |  |  |
| GB34 (Yang Ling Quan) |  | V |  |  |  |  |  |  |  |  |  | V |  |
| TB3 (Zhong Zhu) |  |  | V | V |  |  |  |  |  |  |  |  |  |
| SI3 (Hou Xi) |  |  | V | V |  |  |  |  |  |  |  |  |  |
| ST38 (Tiao Kou ( |  | V |  |  |  |  |  | V | V |  |  |  | V |
| BL57 (Cheng Shan ( |  |  |  |  |  |  |  | V |  |  |  |  |  |
| BL1 (Jing Ming) |  |  | V |  |  |  |  |  |  |  |  |  |  |
| LI20 (Ying Xiang) |  |  |  | V |  |  |  |  |  |  |  |  |  |
| TH23 (Sizhukong) |  |  |  | V |  |  |  |  |  |  |  |  |  |
| SI19 (Ting Gong) |  |  |  | V |  |  |  |  |  |  |  |  |  |
| Zhongping |  |  |  |  |  |  |  |  |  |  | V |  |  |
| Jianqian |  |  |  |  |  |  |  |  |  | V |  |  |  |
| Lingxia |  |  |  |  |  |  | V |  |  |  |  |  |  |

The acupuncture points used in each study. V = the acupuncture points was used.

**Supplementary Table 6.** GRADE recommendations for level of certainty in a meta-analysis.

**Question**: Acupuncture and EA compared to PT and Western medicine for FS

| **Certainty assessment** | | | | | | | **№ of patients** | | **Effect** | | **Certainty** | **Importance** |
| --- | --- | --- | --- | --- | --- | --- | --- | --- | --- | --- | --- | --- |
| **№ of studies** | **Study design** | **Risk of bias** | **Inconsistency** | **Indirectness** | **Imprecision** | **Other considerations** | **acupuncture and EA** | **control** | **Relative (95% CI)** | **Absolute (95% CI)** |  |  |
| **VAS score 0-12 weeks MA** | | | | | | | | | | | | |
| 4 | randomised trials | very serious ^a^ | not serious | not serious | serious ^b^ | none | 85 | 84 | - | MD **1.47 lower** (1.87 lower to 1.07 lower) | ⨁◯◯◯ VERY LOW | CRITICAL |
| **VAS score 1.5-3 months MA** | | | | | | | | | | | | |
| 3 | randomised trials | very serious ^a^ | not serious | not serious | serious ^b^ | none | 55 | 54 | - | MD **1.52 lower** (1.94 lower to 1.11 lower) | ⨁◯◯◯ VERY LOW | CRITICAL |
| **VAS score 4-6 weeks MA** | | | | | | | | | | | | |
| 3 | randomised trials | very serious ^a^ | serious ^c^ | not serious | serious ^b^ | none | 55 | 52 | - | MD **1.36 lower** (2.3 lower to 0.43 lower) | ⨁◯◯◯ VERY LOW | CRITICAL |
| **VAS score 1-3 months EA,MA** | | | | | | | | | | | | |
| 4 | randomised trials | very serious ^a^ | serious ^c^ | not serious | serious ^b^ | none | 79 | 77 | - | MD **1.11 lower** (2.01 lower to 0.21 lower) | ⨁◯◯◯ VERY LOW | CRITICAL |
| **Active external rotation ROM 6 weeks** | | | | | | | | | | | | |
| 2 | randomised trials | very serious ^a^ | not serious | not serious | serious ^b^ | none | 27 | 25 | - | MD **6.61 higher** (4.23 lower to 17.45 higher) | ⨁◯◯◯ VERY LOW | IMPORTANT |
| **Active flexion ROM 6 weeks** | | | | | | | | | | | | |
| 2 | randomised trials | very serious ^a^ | not serious | not serious | serious ^b^ | none | 27 | 25 | - | MD **16.7 higher** (2.85 higher to 30.55 higher) | ⨁◯◯◯ VERY LOW | IMPORTANT |
| **Active abduction ROM 6 weeks** | | | | | | | | | | | | |
| 2 | randomised trials | very serious ^a^ | not serious | not serious | serious ^b^ | none | 27 | 25 | - | MD **17.5 higher** (0.85 lower to 35.85 higher) | ⨁◯◯◯ VERY LOW | IMPORTANT |
| **CMS score 1.5-3 months EA,MA** | | | | | | | | | | | | |
| 3 | randomised trials | very serious ^a^ | not serious | not serious | serious ^b^ | none | 65 | 74 | - | MD **4.08 higher** (3.36 higher to 4.81 higher) | ⨁◯◯◯ VERY LOW | IMPORTANT |
| **CMS score 1.5-2 months MA** | | | | | | | | | | | | |
| 2 | randomised trials | very serious ^a^ | not serious | not serious | serious ^b^ | none | 41 | 51 | - | MD **4.11 higher** (3.37 higher to 4.84 higher) | ⨁◯◯◯ VERY LOW | IMPORTANT |

**CI:** Confidence interval; **MD:** Mean difference

**Explanations**

a. problems randomization, allocation and blinding risk of bias

b. Low sample size in the analysis.

c. Significant heterogeneity in the analysis I2 ≥50.

(GRADEpro GDT: GRADEpro Guideline Development Tool [Software]. McMaster University, 2015 (developed by Evidence Prime, Inc.). Available from gradepro.org.)
